# Investigating the contributing factors to HIV/AIDS infection from the perspective of HIV-infected patients

**DOI:** 10.1186/s40359-021-00513-w

**Published:** 2021-01-25

**Authors:** Morteza Mehraeen, Mohammadreza Heydari, Kamran B. Lankarani, Hassan Joulaei, Marjan Faghih

**Affiliations:** 1 Shiraz HIV/AIDS Research Center (SHARC), Shiraz University of Medical Sciences, Shiraz, Islamic Republic of Iran; 2 Health Policy Research Centre, Shiraz University of Medical Sciences, Shiraz, Islamic Republic of Iran; 3 Department of Biostatistics, School of Medicine, Arak University of Medical Sciences, Faculties Building, Payambar A’zam, University Campus, Baseej Square, Sardasht, Islamic Republic of Iran

**Keywords:** HIV/AIDS, Causality, Attitude

## Abstract

**Background:**

People with HIV have always faced stigma and discrimination. Given the numerous papers that have addressed the psychological and social risk factors in spreading HIV, a pressing question is whether individuals’ mere careless and behavioural flaws can still account for the spread of HIV. Barriers and opposing politic made a hard position for HIV and sex education in Iran.

**Methods:**

The present study investigated the causes of contracting HIV/AIDS from the perspective of HIV-infected patients. To accomplish this, 150 patients referring to the voluntary counseling and testing Center, Shiraz were convenient selected based on the convenient sampling method and responded to a researcher-made questionnaire From June to August 2019. The data were analyzed through descriptive statistics (mean, SD, frequency tables) and inferential statistics (chi-square).

**Results:**

Results revealed that the main cause of HIV infection amongst males was the injection of narcotics, and in the females it was sexual intercourse with an infected individual. Meanwhile, 57% of the females and 66% of the males blamed themselves for contracting and transmitting the disease. The patients stated that if they could return to pre-infection period, they would use one of the following ways to prevent the disease: (a) they would pay attention to hygienic/sanitary principles; (b) they would not get married; and (c) they would prevent drug addiction. Also only 44% of the individuals had successful siblings (those who were neither addicts nor HIV/AIDS-infected individuals), which was an observation that emphasizes on the epidemic of high-risk behaviors in the patients’ families.

**Conclusions:**

According to participants' statements collected in our study, weakness in governmental public health education, along with family-related and individual factors, are important causes of HIV spread

## Background

After the first known case of HIV, discovered in 1981, a wide-ranging line of research was initiated that addressed various dimensions of the disease, such as its etiology, pathogenesis, and psychological aspects [1]. Since there are several ways to contract the disease, HIV-infected individuals always faced with finger pointing, and as of now no definitive treatment has been proposed yet, and that is the reason why such patients, especially unknown cases, are considered to be potential risks for the society. Infected people always face stigma/discrimination and sometimes social rejection [2]. In many counties, HIV is still seen as a divine punishment for promiscuous people, while HIV is regarded as a consequence of ignoring moral principles, consuming drugs, and homosexuality. In some cases, healthy individuals’ inadequate information about the disease discourages them from even relation with HIV-positive people [3]. But since the main cause of HIV has been identified as social harms [4], we can pay attention to much broader issues such as society factors, government politics, education system, health system, and so on. The more people are aware of HIV/AIDS, the less they will fall into it and the more positive their outlook will be [5]. For example we can point to the case of Ryan Wayne White. He was a hemophilic teenager that victim of people’s lack of information about HIV. When the parents of his classmates found out that he had HIV, they forced the school to expel him from school. He was out of society for all of his life. After his death, his parents founded the Ryan White CARE Act to raise public awareness about HIV [6].

Also in the field of HIV education in Iran can be said that, sex and HIV education is associated with strongly opposed in politics and families culture. For this reason, most HIV-related education is metaphorical and less direct.

So if one reviewed HIV infection realistically and profoundly, one would realize that in many cases individuals did not have an active role in catching the disease. When HIV-infected individuals are identified, they encounter numerous challenges in their social interactions such as being labeled or rejected by the society. Studies have shown that such individuals are victims of “hostile views” worldwide [7]. Some studies suggest that individuals who live in regions with a high frequency of risky behaviors are more exposed to HIV infection. Some studies emphasize on the fact that society and the environment are amongst the major causes of infection [8, 9]. Meanwhile, support groups have an important role in preventing AIDS spread, and individuals who enjoy family support pay more attention to preventive behaviors [10, 11]. In all of the mentioned studies, the researchers made an attempt to investigate and highlight the factors affecting individuals’ infection. However, probing into how infections occur and what factors are more effective in the process, from the perspective of patients and their medical records, remain an unexplored topic. Because many studies have focused on the social harms of HIV, it has been assumed that patients also blame social factors. So the present study seeks to focus on the views of HIV-infected individuals and to identify factors affecting or contributing to their HIV infection, based on such categories as individual aspects, society, family, and government.

## Methods

This study was a cross-sectional research which drew on a mixed Quantitative and qualitative design and included 150 patients with HIV who were convenient selected from Shiraz Voluntary Counseling and testing Center (VCT) From June to August 2019. The sample size was decided according to the findings of other studies in the literature [12), and it was confirmed by a statistician at a 65% confidence coefficient.

Because the number of patients who refer to the VCT is small, the researchers had to interview and question all those who had been under treatment by HAART drugs for at least one year and who wanted to participate; regardless of their age, gender and other demographic characteristics.

All the participants filled in the ethics informed consent form of Shiraz University of medical sciences. The exclusion criteria were submitting an incomplete questionnaire or unwillingness to participate. It should be noted that the participants also answered a standard questionnaire of adherence to treatment and a standard questionnaire of mental health and the questions from which this study was extracted were part of their interview.

The data collection tool utilized in this study was a researcher-made questionnaire and the items were selected through brainstorming and using the experts’ opinion. These items were the most probable causes of or contributors to HIV/AIDS infection. The result was a semi-structured interview with 4 questions. The focus of all the questions was on who the patients blamed for their HIV infection. The survey, which included two questionnaires and an interview, took about half an hour. To measure the reliability of the Four-question questionnaire, 20 copies were completed in a pilot study, and its Cronbach’s alpha was 0.82. The validity of the questionnaire was tested through content validity ratio (CVR) based on the opinions of three experts in the field, and the necessary corrections were implemented in the final version of the questionnaire.

Some other required pieces of information were collected through open interviews which addressed the reasons for the success of the patients’ siblings. Success means having attained wealth, position, honors, or the like. In the text of the interview, this goal was marked with a question, but in the interview, sometimes up to 5 questions were asked of the interviewee. The questionnaire and interviews were completed by an expert (with a Master’s Degree) in educational psychology. The data collected from the questionnaires and the interviews were analyzed in SPSS. The analysis of the data included descriptive statistics (frequency table, mean, standard deviation) and inferential statistics (chi-square) we used of Shapiro Wilk for normality and used nonparametric tests where there was no normality data. For analysis of qualitative data used of manual analysis. All the interviews had wrote.

## Results

Demographic characteristics of all patients are shown in Table 1. The statistics showed that 73.6% of females’ infection contracted the diseases from a sexual partner, while only in 1.5% of the cases the males were infected through their spouses or partners. In 82.8% of the cases, the males were infected by injection, whereas in 4.5% of the females HIV was contracted through injection. No case of infection as a result of blood transfusion, medical intervention, or tattooing was observed. In terms of the way the participants were infected, no significant difference was observed amongst different ethnicities under investigation. Based on the findings, those infected by injecting narcotics had a better economic level compared to those who contracted HIV from their spouses or partners, and this difference was statically significant (p = 0.007). Because almost all of the participants that infected by injection were man and almost all of the participants that infected by sex were woman.

**Table 1:** Demographic information

	N (%)	Mean ± SD; min–max
*Sex*		
Male	58(38.7)	43.5 ± 9.6; 10–73
Female	91 (60.7)	40.6 ± 7.05; 15–60
*Age*		
10–20	2 (1.3)	41.69 ± 8.21
21–30	7 (4.7)	
31–40	51 (34)	
41–50	74 (49.3)	
51–60	13 (8.7)	
> 60	2 (1.3)	
*Province*		
Fars	118 (78.66)	
Not fars	32 (21.3)	
*Religion*		
Muslim	147 (98)	
Non-Muslim	3 (2)	
*Education*		
Illiterate	4 (2.68)	
Primary education	116 (77.85)	
Diploma	23 (15.33)	
Academic	6 (4)	
*Education place*		
Shiraz	135 (90)	
Out of Shiraz	15 (10)	
*Reason of leaving education*		
Lack of interest	56 (40.87)	
Lack of possibilities	50 (36.49)	
Lack of support	16 (11.67)	
Unforeseen circumstances	15 (10.94)	
*Married status*		
Single	23 (15.33)	
Married	70 (46.66)	
Divorced	57 (38)	
*How to get infected*		
	Man	Woman
Sex	2 (3.4)	1 (1.1)
Injection	48 (82.8)	2 (2.2)
Tattoo	1 (1.7)	0 (0)
Wife/husband	1 (1.7)	67 (73.6)
Mother child	2 (3.4)	2 (2.2)
Don’t know	4 (6.9)	19 (20.9)
*Independent of family*		
Yes	103 (68.7)	
No	44 (29.3)	
Missing	3 (2)	
*Family size*		
Small (1–3 child)	16 (10.7)	
Medium (4–10 children)	105(70)	
Large (> 10 children)	29 (19.3)	
*Birth order*		
First	40 (26.6)	
Meddle	73 (48.6)	
Last	37 (24.6)	
*Parents*		
Present	43 (28.7)	
Absent	107 (71.3)	
*Parent’s job*		
	Father	Mother
Self-employee	105 (70)	1 (0.7)
Personnel	15 (10)	1 (0.7)
No job	25 (16.7)	0 (0)
Military	3 (2)	0 (0)
Housewives	0 (0)	147(98)
*Parent's education*		
	Father	Mother
Illiterate	70 (46.7)	107 (71.3)
Primary education	72 (48)	41 (27.3)
Diploma	7 (4.7)	2 (1.3)
Academic	1 (0.7)	0 (0)

### Open questions information

Question 1: Which of the following factors had a more effective function in your HIV infection (you can choose more than one alternative)?

The findings showed that 38.9% of the patients blamed themselves for their disease while 13.42%, 20.8%, 2.68%, and 35.7% blamed their families, the society, the government, and their spouses, respectively. Meanwhile, 3.35% blamed other factors.

Among the factors considered for women, "spouse" had the greater role. 17 of participants selected more than one factor contributing to their infection. Yet, the responses mostly emphasis roles of the individuals themselves and their spouses.

Also men more than women, found themselves more effective in their infection (66%), while the females mostly believed to their families and spouses role (57%) and this difference was statistically significant (p ≤ 0.05). Those who were brought up in families without parents did not blame themselves or their spouses and emphasized on other factors. The participants who lived independently (meaning the children who did not live with their parents although might have financially relied on them) did not found themselves or their spouses as effective factors and instead selected other factors. Meanwhile, 28% of the singles, 90% of the married ones, 57% of the divorced ones, and 76% of the widows/widowers, and all temporarily married wives lived independently.

The participants born to a family with 1 child or more than 4 children blamed themselves less than those who were born to families with 2–4 children and this difference was significant (p ≤ 0.05). The patients who were in the middle child in terms of birth order tended to be more HIV/AIDS-infected than those who were the only child, the first child, or the last child. As mentioned, only one participant was infected as a result of a homosexual intercourse. Mother-to-child transmission of HIV occurred in the case of the patients who were the only child, the first child, or the last child, and in the case of middle children mother-to-child transmission of HIV was not observed; from a sexual perspective; hence, no difference was observed. Also males more than the females, were more aware of how they contracted the disease.

Question 2: Determining the contributing factors.

The responses to the above question did not reveal any difference between number of “myself” or “society” and ethnicity, education level, and siblings. Yet, the married participants, more than the single ones, tended to find themselves and the society more effective. Also no differences between the choice “family” and any of the demographic items.

As Table 2 shows, most of the participants had a poor economic status with an expenditure rate of IRR 7,000,000–12,000,000. The chi-square test revealed the difference between the responses of the two genders with poor economic status to such items as “myself”, “society”, “family”, and “government.” In the participants with a moderate economic level, “family” was a significant factor and in those with good economic status, responses to items of “society” and “government” were significant (p ≤ 0.05). Furthermore, the difference between the effective factors selected and economic status was significant in terms of “myself”, “society”, and “family” (p ≤ 0.05). It should be noted that each participant could choose more than one choice  and the financial findings dates back to the financial status before the 2018 inflation. Also considering that the participants could add any factor in response to the “Other Factors” section, all of the females mentioned “spouse” but the males wrote different factors such as co-workers, bad friends, etc.

**Table 2:** Association between patient answers and their economy level

Answer	Sex	Effectiveness								
		8–10			4–7			0–3		
		The economic situation			The economic situation			The economic situation		
		Average upward	Average	Low	Average upward	Average	Low	Average upward	Average	Low
Myself	Female	**76**	**7**	**0**	2	4	0	2	0	–
	Male	**4**	**2**	**3**	5	4	2	29	9	–
Family	Female	**68**	**11**	**0**	**5**	**0**	**0**	7	–	–
	Male	**36**	**14**	**4**	**0**	**1**	**1**	2	–	–
Society	Female	**66**	**8**	**0**	3	2	0	**11**	**1**	**0**
	Male	**34**	**14**	**3**	3	1	1	**1**	**0**	**1**
Government	Female	**78**	**10**	**0**	1	1	–	1	1	–
	Male	**37**	**15**	**5**	0	–	–	1	0	–
Others	Female	31	5	0	3	1	–	**46**	**5**	**–**
	Male	38	14	5	0	0	–	**0**	**1**	**–**
Total		468	100	20	21	13	4	100	17	1

The Bold words and numbers point to a statistical significance in terms of gender

The participants were divided into four categories, according to their monthly income: low (IRR 0–6,990,000), moderately low (IRR 7,000,000–9,990,000), moderate (IRR 10,000,000–19,990,000), and moderately high (more than IRR 20,000,000). The individuals with a low or moderately low economic status expressed risky sexual behaviors more than the individuals with a moderate or moderately high status (p ≤ 0.05). There was a significant and inverse association between the income of families and the scores of the individuals who found their families and the government effective in their infection. That is to say, the individuals coming from families with a lower income rate tended to highlight “family” and then “government” as factors contributing to their infection. Yet, economic status did not have any impact on preventive measures before the individuals were infected. Moreover, there was no significant association between the presence of other successful siblings in a family and the economic status of the infected individuals.

Question 3: What preventive measures would you take if you could return to your pre-infection period?

In response to the above question, 53.4% of all participants, 63.23% of those who contracted HIV through their spouses or partners, and 35.29% of those who were infected by injection stated that if they could return to their pre-infection period, they would observe hygienic/sanitary principles to prevent the disease. More specifically, they would use condoms or other effective tools that could prevent the transmission of the disease, and they would be more careful about their genital hygiene. Meanwhile, 15.8% of all participants, and 30.88% of those who contracted HIV through their spouses, emphasized that they would never get married; 20.3% of all participants and 51% of those who were infected by injection stated that they would prevent HIV by preventing addiction. Yet, 9.3% and 11.3 of all participants did not express any ideas (“No Idea”) or totally ignored this question, respectively. The individuals who stated they would prevent addiction tended to find themselves more effective in their infection.

Question 4: Was there any successful person in your siblings (brothers/sisters)?

In response to the above question, 44% of the participants mentioned that there were successful individuals in their family. The number of successful sisters and successful brothers in such families were almost equal. The designated successful people were engineers, employees, military personnel, clergymen, cultural workers, medical workers (e.g. nurses, operating room technicians, and pharmaceutical companies’ employees), and drivers.

Question 5: If there is a successful person in your family, what factors do you think contributed to their success and why such factors did not apply to you?

The participants enumerated 10 factors that helped their siblings achieve success in their lives; the factors were divided into three categories, namely individual, social and familial: (a) individual: having good life skills, awareness of diseases and their ways of transmission, pursuing their education up to higher levels, having a good financial status after marriage, living with no addiction, having negative views about drugs; (b) social: avoiding bad friends, a healthy and secure environment; and (c) familial: family support and compassion, a healthy and secure environment, a suitable marriage.

## Discussion

The factors contributing to an individual’s infection might be controllable or might be beyond his/her control. To understand the circumstances that could help an individual to prevent HIV/AIDS, this study attempted to focus on the uncontrollable factors; one of the important ones was *forced marriage*. Most of the females who were married under duress stated that they had limited information about how the diseases could be transmitted and how to prevent it; meanwhile because they were not aware of the fact that their husbands were infected, the females, more than males, tended to blame other factors (particularly their spouses) and then their families for their infection. One reason for this tendency was that such females, who were forced by the families to get married, were infected by their husbands who had contracted HIV/AIDS through injection.

Fallahzadeh et al. (2009) and Haghdoost et al. (2011) showed that Iranian women in most cases contracted HIV/AIDS through sexual intercourse with their spouses; this observation was in line with the findings of the present study [13, 14]. This condition may evoke a sense of indignation and injustice in the infected individual and lead to the breakdown of family, child neglect, and the family’s inability to educate or support children [15]. Since almost all of the females came from low social class, lived in slums, or were immigrants, they were most probably victims of marriage under duress. Given this premise, it is understandable as to why they blamed their families. Field studies have emphasized that one of the most effective ways in preventing communicable diseases and controlling addiction is employment for males; naturally the government is responsible for creating jobs, although in the cases under investigation the females also blamed the government [16].

The study revealed that the family’s failure to support and disregard to children were an effective factor in leading them toward risky behaviors; about 90% of the patients were brought up in large or medium-sized families. Because their parents were not able to sufficiently take care of their children, it could be argued that most of the patients had abusive parents, and this factor was beyond the patients’ control. The individuals who were raised in high-stress environments and developed risky behaviors mostly blamed factors other than themselves in their infection; in contrast, those who were brought up in small families mostly found their parents and society effective in their infection.

As these individuals had a specific position in their families, they had natural expectations of the environment and found others effective in their HIV infection. This issue not only highlights the importance for the presence of parents in a family, but also it underscores how a large number of children in a family, willful parental absence, or parental death could affect children’s sense of responsibility. This observation was in line with the findings of studies that showed interpersonal relations in the family and parental treatment could help to prevent risky and delinquent behaviors [17–19].

Some researchers believe that individuals who are raised as the only child in the family are given special care by their parents and tend to place responsibility on other people’s shoulders; in contrast, individuals brought up in very large families, where parents are not capable of responding to even children’s primary needs, tend to find *others* responsible for their problems in life [20].

Another question concerns birth order and how it could affect risky behaviors. This study revealed that children born in the middle tend to fall victim to risky behaviors more than those born as the first child, the only child, or the last child. One reason for this possibility is that parents usually pay more attention to the first child, the only child, or the last child, although this is not the only reason. Alfred Adler contended that first born children, due to their relative self-sufficiency, and last children, because of their tendency to stay with their families, construct a more conservative pattern in their behaviors and actions, especially first born children who have to play the role of parents very early in life for their siblings [21]. Therefore, middle children tend to show risky behaviors as they usually receive less parental attention and live in a multi-care conditions. This observation is further supported by the association between birth order and ways of infection.

Furthermore, the results of this research clarified that the females were less aware than the males on how they contracted HIV. The fact that 53% of the participants stated they would observe personal hygiene (using condoms mostly) if they could return to their pre-infection period revealed that the patients were not sufficiently aware of AIDS. This observation, however, was in contrast with the findings of Noroozi and et al. (2017), who reported that the people who inject drugs in Kermanshah had a good or excellent level of understanding HIV/AIDS [22]. Yet, Ramezani Tehrani and Malek-Afzali’s (2008) findings of their research into the high-risk population of Tehran were mostly in line with the observations of the present study [23]. The reason that can account for the difference observed in Montazeri’s study is the type of population under investigation (high-risk population versus general population). Of course an individual might get infected because of his/her lack of tendency to gain information about the diseases, which was not explored in the present study.

Another observation was that living in the existing conditions was not a serious concern for at least half of the participants (47%) as they probably could not think of any better conditions. As a result, if they could return to their pre-infection period they would most probably get married with the same individuals. In such cases, more effective health system and more active media could be helpful by raising people’s awareness of the disease and reduce their probability of infection. Yet, the patients did not blame the government for their lack of awareness. In 63% of the statements, the participants suggested that if they were aware of the ways of HIV infection, they would have observed hygienic principles. Furthermore, apart from 20% of the participants who stated they would prevent addiction to prevent infection, the rest of the patients who contracted the disease through injection did not regret their addiction.

It was also found that the participants who would care about hygienic principles if they could return to their pre-infection condition, did not find themselves much effective in their infection. In contrast, the participants who would prevent addiction to prevent the disease, if they could return to their pre-infection condition, found themselves more effective in their infection. Moreover, those who stated they would totally avoid marriage to prevent infection did not blame themselves at all for their infection. This situation revealed that there was a significant association between how the patients contracted the disease and how they would prevent it by blaming oneself or others.

The economic conditions of the patients revealed that 96.7% of the infected individuals had a low or moderately low economic. Such individuals, as opposed to those having moderate or moderately high income, tended to engage in high-risk sexual activities; this finding was in line with that of de Souza et al. [24]. Considering the fact that one of the major responsibilities of a government is to generate jobs and provide financial means, the findings showed that the individuals coming from low or moderately low socioeconomic families found the government and then family as the most important factors contributing to their infection. Nonetheless, as economic status was not significantly associated with the individuals’ preventive tendencies before infection, the individuals would practice preventive measures under any economic conditions. This tendency again emphasized on the role of education and the improvement of individuals’ preventive skills in the case of HIV/AIDS.

The non-significant association between economic status and successful siblings in the family, as well as the reasons mentioned for the success of the siblings, emphasized that even under harsh economic conditions, people with effective individual/social skills (e.g. life skills, a suitable marriage, friend-finding skills, or other characteristics mentioned by the patients as their siblings’ success factors) could manage to achieve success, despite difficulties and avoid family-related problems. Of course, governmental intervention must be exercised in various fields of education including individual, familial and social; such intervention must address planning and procurement of educational materials at all levels ranging from elementary training (kindergartens) to higher education or other social strata.

Although there was not a significant association between economic status and preventive factors, individuals with a low or moderately low economic status tended to show more high-risk sexual behaviors than those with a moderate or high-moderately high status. This condition highlighted that the government’s role, while investing in the training of basic life skills, must seriously address employment, especially for young people and those eligible for work; in this process individuals with poor personal or familial economic conditions must be prioritized, so that high-risk sexual behaviors can be controlled.

This study, clearly had some limitations; first; sample size of the participants was small (150 participants) for the study due to financial limitations. Secondly, because participants had to answer several pages of interviews and questionnaires; in some cases the long length of the interviews was boring to the participants and some even left during the interview.

## Conclusion

The finding of this study was that socio-economic factors contributing to a reduction of harm must be specifically taken into account. To accomplish this, the government, public participation, and publicly regulated plans must be prioritized and implemented. Immigrants residing in slums are more vulnerable to the spread of diseases (e.g. HIV/AIDS). These areas must be seriously considered in state-governed plans. Based on the findings of this study, most of the participants claimed that they would pay more attention to preventive measures, if they could return to their pre-infection conditions. This tendency emphasizes on the role of health system in training life skills, tolerance, and other preventive practices that could help to avoid infection. This goal can be practically accomplished by the State Health and Treatment Network System.

Another important issue is the effective function of screening tests in high-risk groups of the population before marriage; in this case, applicants can simply opt in. Finally, as the results showed, many of the females stated that they surrendered to marriage under duress urged by their families, due to their specific life conditions, and their marriage ultimately led to their infection through their infected spouses. This observation underscored the importance of establishing social and legal support plans that could seriously and comprehensively help women to enjoy more economic, legal and social support, especially in the case of vulnerable girls and women. Furthermore, given the females’ high rate of partner infection and the females’ lack of awareness of their spouse’s infection, plans must be formulated to administer optional pre-marriage tests.

In the end can say that the hypothesis of this article was confirmed because even men that stated they were to blame in infection to HIV, said that family situation and friends had an important role in their sickness.

## Data Availability

The datasets during and/or analysed during the current study available from the corresponding author on reasonable request.

## Abbreviation

HIV: Human immunodeficiency virus
